# China’s Outward Direct Investment in the United States: From the perspective of agglomeration economies

**DOI:** 10.1371/journal.pone.0269602

**Published:** 2022-06-10

**Authors:** Xinyi Liang, Junsong Wang, Bingquan Lin, Xianzhong Cao, Yuefang Si

**Affiliations:** 1 The Center for Modern Chinese City Studies, East China Normal University, Shanghai, People’s Republic of China; 2 Department of Geography, Hong Kong Baptist University, Hong Kong, People’s Republic of China; 3 School of Urban & Regional Sciences, East China Normal University, Shanghai, People’s Republic of China; DePaul University, UNITED STATES

## Abstract

Over most of the last two decades, China’s Outward Direct Investment (CODI) has reshaped the global economic landscape and attracted considerable attention. Although extensive research shows that CODI features agglomeration, there is limited research from the perspective of different patterns of agglomeration economies at the subnational level. It is unclear which patterns of agglomeration economies play a role in the location choice of CODI, especially with the variations of CODI in terms of entry mode and ownership. Therefore, based on the data of the CODI in the United States in the period 2000–2016, we use a conditional logit model to investigate the influence of specialized and diversified agglomeration of local firms as well as industry-specific and industry-diverse agglomeration of Chinese investors on the location choice of CODI, and further explore the heterogeneous influence concerning the entry mode and ownership. Our results show that among a variety of agglomeration economies, the specialized agglomeration of local firms is the premier factor influencing the location choice of CODI, even exceeding the influence of industry-specific agglomeration of CODI in the same industry. Industry-diverse agglomeration of CODI plays a weak role, while diversified agglomeration of local firms has no effect. Moreover, the location choice of acquisition is more sensitive to the specialized agglomeration of local firms than that of greenfield investment, and the influence of the industry-diverse agglomeration of CODI has no effect on the location choice of acquisition. In terms of the ownership, the location choice of state-owned enterprises (SOEs) is more sensitive to the specialized agglomeration of local firms and industry-specific agglomeration of CODI than that of private investment, and the industry-diverse agglomeration of CODI has no significant impact on the location choice of SOEs’ offshoring subsidiaries.

## 1. Introduction

Driven by the “Go Global” policy initiated in 1999, CODI has grown quickly over most of the last two decades. By the end of 2019, CODI stock had reached $2198.88 billion, increasing from $2.99 billion in 2002 and accounting for 6.4% of the global stock, third only to the United States and the Netherlands. Against this background, scholars have been extensively exploring the drivers, entry modes, and location choice of CODI [1, 2]. Despite the fact that CODI is mainly concentrated in the United States from the country level, as well as the heated discussion concerning the trade and investment relationship between China and the United States, the research on the location choice of CODI in the United States is mostly incorporated into that of CODI in developed countries in general [3], which cannot reflect the specific mechanism of the location choice of CODI in the United States.

The location choice of CODI has received significant attention from scholars, since it is often regarded as a big challenge for Chinese multinational enterprises to face the huge liability of foreignness when investing abroad [2, 4]. Under such circumstances, scholars found that agglomeration economies wield significant influence on the location choice of CODI [5–7]. Given the limited knowledge of foreign markets and lack of competitive advantages in technology and management, the accessibility of more information is crucial to the location choice of CODI when entering a “primitive” market [8]. To decrease uncertainties and attain agglomeration externalities, Chinese investors tend to locate where the local firms agglomerate or follow the existing location decision of CODI [5, 8]. Although the influence of agglomeration economies on the location choice of CODI at the country level has been verified [9, 10], there has been little attention paid to the subnational level analysis. This is a significant omission given that knowledge spillovers occur locally and regional heterogeneity is regarded as the main factor in determining the specific investment location of multinational enterprises [11, 12]. With respect to the location choice of CODI in the United States, the small amount of research has mainly focused on the location advantages, while little attention has been paid to the role of agglomeration economies at the subnational level [13]. Furthermore, there is a consensus among scholars that entry mode and ownership influence the location choice of CODI [14–16].

Therefore, this study intends to use a conditional logit model based on the data from the US-China Investment Project to investigate the role of agglomeration economies for CODI in the United States at the regional level and to further explore the heterogeneous influence concerning the entry mode and ownership. More precisely, this study investigates whether the specialized or diversified agglomeration of local firms and the industry-specific or industry-diverse agglomeration of CODI can influence the location choice of CODI. In terms of research period, because CODI in the United States was rare and negligible before 2000 and the pace of CODI in the United States has slowed sharply since the inauguration of Donald Trump in 2017, this study selects the period between 2000 and 2016.

The remainder of the study proceeds as follows: In the next section, we conduct a literature review and propose hypotheses based on this. The data of CODI in the region of the United States and the methodology employed to test the hypotheses are clarified in section 3. Section 4 presents and explains the main results. The final section contains the conclusion and discussion.

## 2. Theoretical reviews

Industrial differences and foreign investors make the patterns of agglomeration economy complicated. Agglomeration economy refers to the benefits generated by firms and individuals being close to each other in a region [17]. The traditional agglomeration economies refer to the advantages arising from the specific industry as suggested by Alfred Marshall, including pools of skilled labour, specialized suppliers, and knowledge inflows from competitors [18]. In contrast to Alfred Marshall, Jane Jacobs argued that diverse and varied industries foster opportunities to imitate, share and recombine ideas and practices across industries, which may create the potential for economic growth [19]. Later, with the expansion and deepening of globalization of multinational enterprises, scholars found that agglomeration economies arose not only from the local firms, but also from foreign investors, especially those from the country-of-origin [20]. In the existing research conducted on the agglomeration economies, there are some distinctions between different patterns of agglomeration economies, which become more prominent after considering the strategy of firms [21]. To clarify the influence of the different patterns of agglomeration economies on the location choice of CODI, we mainly divide agglomeration economies into specialized and diversified agglomeration of local firms, as well as industry-specific or industry-diverse agglomeration of Chinese investors.

### 2.1 Specialized agglomeration or diversified agglomeration of local firms

For Chinese investors, agglomeration economies arising from local firms are important for their location choice. In order to maintain normal operations abroad, considering the unfamiliarity with culture, regulations, and competition in the host market, foreign investors need to pay the additional costs that local firms would not incur, known as “liability of foreignness” [4, 22]. Moreover, the liability of foreignness increases when foreign firms lack the strength of ownership advantages relative to their competition in the host country [23]. For example, because of inexperience in international markets and limited ownership advantages (such as weaker marketing resources and technology resources), CODI face a heavy liability when they enter developed countries that constitute a completely distinctive institutional and social environment [5, 24]. In this case, locations that can provide more information become foreign investors’ preferred place to invest [25]. Agglomeration economies are conducive to reducing information asymmetry, since foreign investors can access knowledge spillovers from the region where local firms aggregate. Local firms’ possession of location-specific advantages relating to the local economy helps foreign investors to overcome barriers in the new market [26]. In addition, with the background of the host-country market, local firms have stronger inter-firm linkages because they hire domestic workers and cooperate with local firms frequently, generating more significant agglomeration economies [27].

Furthermore, the influence of agglomeration economies in the United States on the location choice of CODI needs to be further distinguished based on the industry. Knowledge spillovers, local market pooling and inter-firm linkages are the three advantages for the formation of agglomeration economies that can help multinationals do business efficiently [28]. The same advantages that give rise to agglomeration economies mean that foreign investors can benefit not only from the specific industry (i.e., Marshallian externalities), but also from the diversity of industries (i.e., Jacobs externalities) [22]. Although existing research indicates that both Marshallian and Jacobs externalities play an important role in the location decisions of foreign firms, it is worth noting that not all firms are subject to the same economies of agglomeration, which thus cannot be expected to have an identical impact [20, 29]. As Ning et al. suggested, specialized agglomeration promotes knowledge spillovers, while diversified agglomeration may booster innovation in a vibrant environment [30]. The influence of diversification agglomeration has been observed amongst ‘mature’ multinational enterprises from developed countries, however, specialized agglomeration seems to align the interests with the strategy of CODI, which is characterized by learning-based knowledge-seeking strategies [3]. Chinese firms’ low technology absorptive capacity may hinder the inter-industry spillovers from a diversified industrial structure within the foreign environment [30]. Under the assumption of strategic-asset-seeking, CODI are more likely to choose regions characterized by specialization agglomeration of local firms to better augment their production and technological capabilities [31]. As Barrios et al. pointed out, high-value-added and innovative firms are prone to first locate in urban centres in order to take advantage of knowledge-related spillovers from the diversity of industries [29]. In contrast, low-tech firms seem to be only influenced by agglomeration economies created by Marshallian externalities. Therefore, the location choice of CODI may tend more towards the region where the local firms of the same industry aggregate. Hence, we propose the following hypothesis:

H1: The higher the degree of specialized agglomeration of local firms in a state, the more likely CODI of the same industry will enter.

### 2.2 Industry-specific or industry-diverse agglomeration of Chinese investors

In addition to the agglomeration economies of local firms benefiting CODI, the agglomeration economies of Chinese investors can also benefit later investors from China. In contrast to the advantages of local firms in terms of local knowledge and resources, foreign firms from the same origin country possess knowledge that may be more targeted to new entrants. Given the same background, new foreign entrants may face similar obstacles with prior foreign entrants from the same country [32]. In this case, foreign firms from the same origin country can provide some certain firm-specific (home-based) resources to enable survival and higher performance in the foreign market [24]. Furthermore, when foreign investors face high liability of foreignness, the knowledge they seek may concern sensitive cultural and institutional aspects, which may not be easily obtained from local firms [21]. In addition, new entrants can find it easier to gain legitimacy when they co-locate with firms from the same origin country because of the same ethnic identity [21]. They can learn from the early entrants from the same origin country not only how to gain legitimacy, but also to build on the legitimacy they enjoy back home. Along this line of reasoning, the agglomeration economies arising from foreign investors from the origin country are important to the location choice of new foreign entrants.

Similar to the classification of agglomeration economies of the host countries, a further distinction needs to be made between industry-specific and industry-diverse agglomeration of Chinese investors. Admittedly, with the same ethnic identity, any kind of agglomeration of CODI can support new entrants from China in accessing the knowledge spillovers [5]. However, because of the industrial differences, industry-specific agglomeration of CODI can provide more industry-specific knowledge and inter-firm linkages for the later entrants from China in the same industry. As Florida pointed out, the co-location of backward- and forward-linked manufacturing enterprises from the same country is a significant factor that plays a great role in the industrial location of multinational enterprises investing abroad [33]. Furthermore, in order to reduce competition and avoid uncertainties from the industry side in the foreign context, firms tend to follow the investment decisions of the same industry pioneer firms [34]. Therefore, we propose the following hypothesis:

H2: The more CODI stock of a specific industry in a state, the more likely it is that CODI of the same industry will enter.

## 3. Data and methodology

### 3.1 Data sources and the description of CODI in the United States

The data for CODI in the United States mainly used is from the US-China Investment Project, which is led by Rhodium Group and the National Committee on US-China Relations in partnership with the American Chamber of Commerce in Shanghai and the China General Chamber of Commerce in the United States. The data set was collected in a bottom-up way, with the original data coming from multiple channels including business service databases, disclosure reports, officially published information, and social media, which means the dataset can provide information that is as complete as possible. Every transaction amount over 1 million dollars was recorded. Specifically, the dataset captures three types of transactions: (1) acquisitions of existing assets that result in at least 10% ownership stakes; (2) greenfield projects with at least 10% ownership stakes (newly built facilities such as factories, warehouses, offices, and R&D centres); (3) the expansion of existing CODI operations. This not only provided flow data and number of investment projects, but also detailed information about the industrial and geographical distribution, entry mode and investor ownership.

Looking back at the history of CODI in the United States, three important time points are the introduction of China’s ‘Go Global’ policy in 1999, the Financial Crisis in 2008 and Donald Trump’s Inauguration in 2017, deeply influencing the historical development of CODI in the United States, and accordingly dividing the historical development into four stages. Although China introduced the ‘Reform and Opening up’ policy in 1978, the first CODI occurred in the United States in 1986. At this stage, CODI in the United States was rare and negligible. The investigation of the data collected by Rhodium Group shows that CODI in the United States during the period 1990 to 1999 was negligible, with only 12 investments totalling 24 million dollars. This is not only because Chinese per capita income was still low at that time, but also because of the Chinese government’s cautious attitude towards CODI approval. Specific analysis of that time reveals that China only invested in eight states, and that New York attracted most of the investment. After the Chinese government implemented the ‘Go Global’ policy in 1999, CODI expanded to more states, with 29 states having accepted CODI by the end of 2007. However, the transaction volume in this period is generally low, and the average annual investment is still less than 160 million. Most CODI was concentrated on the coasts in California and New York, as well as a few other states hosting large firms or investments, including North Carolina, Michigan, and Texas.

2008 was the beginning of stage 3, not only because of the turbulent international economic situation caused by the financial crisis, but also due to the officially launched BIT negotiations between China and the United States. The financial crisis hit the United States economy hard, which led to its increasing emphasis on foreign direct investment, especially from emerging markets. At the same time, CODI maintained rapid growth while other countries reduced investments. During this period, CODI flow into the United States increased dramatically, from $771 million in 2008 to $46.215 billion in 2016, with an average annual increase of nearly 59%. In terms of the geographical distribution, the scope of investment covers almost the entire United States. By the end of 2016, Chinese firms had investments in 46 of the 50 states. Since the inauguration of Donald Trump in 2017, the pace of CODI in the United States has slowed sharply. According to the report *Two-Way Street*: *2019 Update US-China Investment Trends*, punitive tariffs, a trade war, tighter capital controls by the Chinese government and increased scrutiny of foreign acquisitions in the United States are among the main reasons why CODI in the United States has plummeted.

Considering the negligible volume of CODI in the United States before 2000 and unpredictable changes after 2017, 2000 to 2016 was chosen as the time span. Table 1 shows the geographical distribution of CODI over the United States during this period. Although CODI spread over 47 states in the United States, when looking at the share of CODI location among states, most of these investments were concentrated in California (23.3%). New York (14.3%), Illinois (8.6%), Kentucky (8.4%), Virginia (8.2%) and Texas (6.5%) also show relatively high regional concentration of CODI. It was also found that the amount of investment is not proportional to the number of investment projects. Some regions, such as Kentucky, Oklahoma, and Kansas, receive fewer projects but a large investment amount, while other regions, such as Washington, New Jersey, and South Carolina, receive many projects but only a small investment amount.

**Table 1:** Distribution of CODI over the states of the United States, 2000–2016.

State name	Investment amount (percent)	Project number (percent)	State name	Investment amount (percent)	Project number (percent)
California	25709 (23.3)	414 (30.5)	Missouri	366 (0.3)	13 (1.0)
New York	15703 (14.3)	120 (8.8)	Colorado	345 (0.3)	10 (0.7)
Illinois	9418 (8.6)	66 (4.9)	Pennsylvania	338 (0.3)	20 (1.5)
Kentucky	9270 (8.4)	5 (0.4)	Alabama	318 (0.3)	12 (0.9)
Virginia	9054 (8.2)	27 (2.0)	Wyoming	285 (0.3)	1 (0.1)
Texas	7100 (6.5)	111 (8.2)	Indiana	280 (0.3)	15 (1.1)
North Carolina	4738 (4.3)	58 (4.3)	New Hampshire	230 (0.2)	5 (0.4)
Michigan	4039 (3.7)	79 (5.8)	Oregon	203 (0.2)	9 (0.7)
Oklahoma	3666 (3.3)	3 (0.2)	Maryland	148 (0.1)	18 (1.3)
Minnesota	2825 (2.6)	11 (0.8)	Utah	90 (0.1)	9 (0.7)
Kansas	2729 (2.5)	3 (0.2)	Delaware	81 (0.1)	7 (0.5)
Massachusetts	2686 (2.4)	32 (2.4)	District of Columbia	72 (0.1)	3 (0.2)
Connecticut	2024 (1.8)	4 (0.3)	Mississippi	60 (0.1)	2 (0.1)
Georgia	1346 (1.2)	37 (2.7)	Arizona	50 (0.0)	4 (0.3)
Florida	1235 (1.1)	23 (1.7)	Iowa	46 (0.0)	3 (0.2)
Hawaii	880 (0.8)	8 (0.6)	Montana	41 (0.0)	2 (0.1)
New Jersey	849 (0.8)	41 (3.0)	Maine	30 (0.0)	1 (0.1)
Washington	683 (0.6)	46 (3.4)	Arkansas	16 (0.0)	6 (0.4)
South Carolina	678 (0.6)	40 (2.9)	Idaho	12 (0.0)	4 (0.3)
Louisiana	512 (0.5)	6 (0.4)	Nebraska	9 (0.0)	4 (0.3)
Ohio	509 (0.5)	32 (2.4)	Alaska	7 (0.0)	3 (0.2)
Tennessee	498 (0.5)	19 (1.4)	West Virginia	5 (0.0)	1 (0.1)
Wisconsin	400 (0.4)	5 (0.4)	Rhode Island	1 (0.0)	1 (0.1)
Nevada	434(0.4)	15 (1.1)	Total	110058 (100)	1358 (100)

From a dynamic perspective, the distribution of CODI from 2000 to 2016 shows a tendency of agglomeration towards the Northeast Corridor and the Midwest. Prior to 2005, China had a small amount of investment in the United States. Only North Carolina became an outstanding state, since a big project was undertaken there by Beijing in the Information and Communications Technology industry through acquisition in 2005. In the period 2006–2010, Chinese firms increased their investment in the Northeast Corridor and the Midwest, and the investment industry was dominated by the energy and automotive industries. After 2011, the expansion of Chinese firms broadened further, including to the Pacific Northwest, the South, and parts of the Midwest, and became more diversified, including not only the traditional resource-based industries, but also emerging industries such as Entertainment, Media, and Education, as well as the Financial and Business Services industry. Taken together, by comparing the three sub-plots, the change of the distribution of CODI in the United States seems to show a trend of agglomeration.

### 3.2 Methodology

The conditional logit model of McFadden [35] was adopted to analyse the location factors of 1358 projects of Chinese investment across fifty-one regions (fifty states and a federal district) in the US during the period between 2000 and 2016. The conditional logit model is derived from profit-maximizing firm behaviour under appropriate assumptions concerning the stochastic term in the profit function, and the model has been widely used in previous empirical studies of location choice. It was assumed that CODI would choose the region that would yield the highest profit with the consideration of a series of attributes of states when deciding where to invest. For every project, CODI could choose any of the fifty-one regions. In this case, if the state was selected, the dependent variable was assigned as 1, otherwise the dependent variable was assigned as 0, which meant that there were many states not selected. In addition, referring to Ben-Akiva et al.’s research, only 5 states that have not been invested in were randomly selected to estimate the model [36]. Since the state that had not been invested in was randomly selected, the result would not be affected. The final estimated sample data was 1358*6.

Regarding the attributes that attract CODI in the United States, two groups of conditional variables were included to estimate the impact of the probability of the project *i* choosing the state *j*. The following model was proposed:

$$P_{ij}=f\left(A_{j},C_{j}\right)$$ (1)

Where *P*_*ij*_ was the project *i* choosing state *j* as its preferred location to maximize utility. *A*_*j*_ represented agglomeration attributes in state *j*, and *C*_*j*_ were control variables for state *j*. The choice model with *n* mutually exclusive alternatives was as follows:

$$P_{ij}=\frac{e^{V_{ij}}}{\sum_{k=1}^{n}e^{V_{ij}}}$$ (2)

Where *V*_*i*_ was a utility function of the explanatory variables related to state *j*. The utility function was determined as follows:

$$V_{ij}=\beta_{0}+\beta_{1}A_{j}+\beta_{2}C_{j}+\varepsilon_{i}$$ (3)

Where *β*_0_, *β*_1_, *β*_2_ were the estimated coefficients and *ε*_*i*_ was an error term. It was hypothesized that the above-mentioned agglomeration economies as well as other attributes of states would affect the location choices of CODI in the United States.

### 3.3 Variables

#### 3.3.1 Whether to invest as dependent variable

Because the model adopted was a conditional logit model, the dependent variable was a dummy variable indicating whether an investment was made in the NUTS 2 area: 1 if yes, 0 if no. The data was from the US-China Investment Project.

#### 3.3.2 Four patterns of agglomeration as independent variables

In accordance with our previous hypotheses, *Local specialized agglomeration*, *Local diversified agglomeration*, *Specialized_CN* and *Diversified_CN* were mainly investigated in the model. The explanations of different patterns of agglomeration were as follows.

In order to capture specialized agglomeration and diversified agglomeration in the states, with reference to Li and Song [37], the relative value to measure was used. If *S*_*jk*_ was defined as the percentage of the output value number of industry *k* in state *j*, the specialization index for the individual state was:

$${ZI}_{j}=max\left(S_{jk}\right)$$ (4)

In order to obtain a horizontal comparison of specialization between different states, the level of relative specialization (rather than absolute specialization) was needed, so the relative specialization index *(Local specialized agglomeration*) was defined as:

$${RZI}_{j}=max\left(\frac{S_{jk}}{S_{k}}\right)$$ (5)

*S*_*k*_ referred to the industry’s share of the country. The most common diversification index to use is the reciprocal of the Herfindahl-Hirschman Index (HHI). The HHI index refers to the sum of the squares of the output value share of all industries. The diversification index measured by its reciprocal was:

$${DI}_{j}=\sum S_{jk}^{2}$$ (6)

The relative diversification index (*Local diversified agglomeration*) was defined as:

$${RDI}_{j}=1/\sum S_{jk}-S_{k}\vee$$ (7)

The output value data of various industries were obtained from the database of the Bureau of Economic Analysis.

The third agglomeration variable (*Specialized_CN*) was the log of the CODI stock in the industry *k* in the United States. The variable captured industry-specific agglomeration of CODI. The measurement of the industry-diverse agglomeration of CODI (*Diversified_CN*) was the log of the CODI stock in the other industries (i.e., not industry *k*) in the United States. The data of CODI was from the US-China Investment Project.

#### 3.3.3 Control variables

Based on a long string of studies on the location choices of CODI, some control variables that might influence the location decision of CODI in the United States were selected. Because of the proximity of the culture and language, overseas Chinese act as an interface and function as localized sources of social and human capital to assist the link-up [8, 38], helping Chinese investors reduce the impediments and risk when investing in the host country. Therefore, the potential influence of the overseas Chinese was controlled. The variable was measured by the proportion of the percentage of overseas Chinese in each state to the percentage of overseas Chinese in the United States, with the data coming from the United States Census Bureau.

In addition to the influence of overseas Chinese, the strategic asset-seeking, market-seeking, and resource-seeking motivations suggested by the theory on the eclectic paradigm were also considered as control variables. Existing studies show that the motivation of CODI in developed countries is mainly strategic asset-seeking [39, 40]. Therefore, CODI in the United States tends towards the states with strong innovation capabilities. The innovation capability of the states was measured by three indicators: patents, R&D expense, and labour quality. The log of the number of patents of the states was used to measure the innovation output of the states. The proportion of R&D expense to the GDP of the states was used to measure the innovation input. Moreover, higher quality of the labour of the states may induce higher innovation efficiency. Therefore, the proportion of the labouring population with a bachelor’s degree was used as the proxy of the labour quality. The data of these three variables were from the National Science Foundation, Science and Engineering Indicators.

Some scholars also found that CODI was driven by the market attraction of developed countries [41, 42]. Therefore, the potential influence of the market-seeking motivation was controlled. The measurement of the market-seeking motivation was the growth rate of the GDP, per capita tax, and per capita income. The data used were obtained from the Bureau of Economic Analysis.

Some Chinese investors may seek natural resources in developed countries through acquisition [43]. Therefore, the proportion of the resource industry to the entire industry of the states was used as the proxy of resource-seeking motivation. The resource industries include forestry, fishing and related activities, mining, as well as oil and gas extraction. The data were from the Bureau of Economic Analysis.

All variable definitions and sources are listed in Table 2. It is worth mentioning that considering the lag of variables, all variables (except the dependent variable) used in the model were from the data of the previous year. In addition, there is no serious multicollinearity problem because the variance inflation factors of the variables are all less than 5. The descriptive statistics of variables are presented in Table 3.

**Table 2:** Variable descriptions and sources.

Variable	Description	Source
*Investment*	Whether to invest in the state: 1 if yes, 0 if no.	US-China Investment Project
*Local specialized agglomeration*	The maximum of the proportion of the percentage of the output value of industry *k* in each state to the percentage of the output value of industry *k* in the United States in the previous year, ${RZI}_{j}=max\left(\frac{S_{jk}}{S_{k}}\right)$.	Bureau of Economic Analysis
*Local diversified agglomeration*	The reciprocal of the sum of the absolute value of the difference between the percentage of the output value of industry *k* in each state and percentage of the output value of industry *k* in the United States in the previous year, *RDI*_*j*_ = 1/Σ|*S*_*jk*_ − *S*_*k*_|.	Bureau of Economic Analysis
*Specialized_CN*	Log of the CODI stock in the industry *k* in the United States in the previous year.	US-China Investment Project
*Diversified_CN*	Log of the CODI stock in the other industries (i.e., not industry *k*) in the United States in the previous year.	US-China Investment Project
*Overseas Chinese*	The proportion of the percentage of Chinese in each state to the percentage of Chinese in the US in the previous year.	United States Census Bureau
*Patents*	Log of the number of patents of the states in the previous year.	*National Science Foundation*, *Science and Engineering Indicators*
*R&D expense*	The proportion of R&D expense to the GDP of the state in the previous year.	*National Science Foundation*, *Science and Engineering Indicators*
*Labour quality*	The proportion of the labouring population with a bachelor’s degree in the previous year.	*National Science Foundation*, *Science and Engineering Indicators*
*Growth_GDP*	The growth rate of GDP of the states in the previous year.	Bureau of Economic Analysis
*Tax*	Log of the per capita tax of the states in the previous year.	Bureau of Economic Analysis
*Income*	Log of the per capita income of the states in the previous year.	Bureau of Economic Analysis
*Resource*	The proportion of the resource industry to the entire industry of the states in the previous year.	Bureau of Economic Analysis

**Table 3:** Descriptive statistics for variables.

Variable	Observations	Mean	Std. Dev.	Min	Max
*Investment*	8148	0.167	0.373	0	1
*Local specialized agglomeration*	8046	1.046	0.989	0	13.446
*Local diversified agglomeration*	8046	4.072	1.531	1.102	10.28
*Specialized_CN*	8046	1.119	1.958	0	8.868
*Diversified_CN*	8046	3.277	2.721	0	9.196
*Overseas Chinese*	7230	0.776	0.895	0.035	6.833
*Patents*	7638	6.976	1.623	2.89	10.609
*R&D expense*	7908	2.33	1.489	0	8.077
*Labour quality*	7104	29.989	6.08	19.91	62.173
*Growth_GDP*	8046	3.685	3.195	-15.3	24.7
*Tax*	7961	7.832	0.33	7.159	9.45
*Income*	8046	10.621	0.201	9.982	11.231
*Resource*	8046	0.044	0.063	0	0.391

## 4. Results

As Table 4 shows, the results confirm the above-mentioned hypotheses. Table 4 presents the results of the econometric model. Specifically, Model 1 contains only the control variables, Model 2 introduces *Local* s*pecialized agglomeration* and *Local diversified agglomeration* and control variables, Model 3 introduces *Specialized_CN*, *Diversified_CN* and control variables, and Model 4 encompasses all the variables. Firstly, through comparing the difference of the Pseudo R2 between the four models, the inclusion of four patterns of agglomeration economies is found to improve the model specification, indicating that the agglomeration economies do influence the location decision of CODI, and the inclusion of agglomeration economies from the US local firms as well as from CODI are crucial in terms of the clarification of the location choice of CODI in the United States. Next, Model 4 shows that *Local specialized agglomeration* is significantly positively related to the *investment*, while *Local diversified agglomeration* has no relation with the *investment*, verifying H1. This result may indicate that knowledge spillovers from specialized agglomeration may better satisfy Chinese knowledge-seeking needs [3].

**Table 4:** Conditional logit estimation for CODI in the United States.

	Model 1		Model 2		Model 3		Model 4	
Variable	Coefficient	Elasticity	Coefficient	Elasticity	Coefficient	Elasticity	Coefficient	Elasticity
*Local specialized agglomeration*	_	_	0.485*** (0.044)	0.475	_	_	0.347*** (0.046)	0.340
*Local diversified agglomeration*	_	_	-0.028 (0.036)	-0.027	_	_	0.014 (0.039)	0.014
*Specialized_CN*	_	_	_	_	0.305*** (0.025)	0.299	0.238*** (0.026)	0.233
*Diversified_CN*	_	_	_	_	0.074*** (0.026)	0.073	0.087*** (0.027)	0.085
*Overseas Chinese*	0.530*** (0.067)	0.520	0.565*** (0.073)	0.554	0.366*** (0.068)	0.359	0.441*** (0.071)	0.432
*Patents*	0.817*** (0.048)	0.801	0.793*** (0.052)	0.777	0.486*** (0.056)	0.476	0.487*** (0.061)	0.477
*R&D expense*	-0.091*** (0.035)	-0.089	-0.131*** (0.037)	-0.128	-0.028 (0.037)	-0.027	-0.058 (0.039)	-0.057
*Labour quality*	-0.013 (0.020)	-0.013	0.004 (0.022)	0.004	-0.030 (0.023)	-0.029	-0.025 (0.024)	-0.025
*Growth_GDP*	0.025 (0.021)	0.025	0.038* (0.021)	0.037	0.019 (0.020)	0.019	0.032 (0.021)	0.031
*Tax*	-0.837*** (0.274)	-0.821	-1.049*** (0.277)	-1.028	-0.896*** (0.292)	-0.878	-1.019*** (0.287)	-0.999
*Income*	-0.969 (0.718)	-0.950	-0.872 (0.790)	-0.855	-0.050 (0.775)	-0.049	0.135 (0.833)	0.132
*Resource*	-2.926* (1.637)	-2.869	-6.124*** (1.709)	-6.004	-4.207* (1.677)		-5.605*** (1.715)	-5.495
Number of observations	7027		7027		7027		7027	
Pseudo *R*^*2*^	0.382		0.4155		0.4283		0.441	
Log pseudolikelihood	-1301.020		-1231.2715		-1204.3067		-1177.289	

Notes: In the practical process of using Stata, we use "robust" command to account for potential model misspecification. Robust Standard errors in parentheses.

*** p<0.01,

** p<0.05,

* p<0.1.

Thirdly, the industry-specific agglomeration and industry-diverse agglomeration of CODI both show a significantly positive correlation with the investment. To further clarify their role, with reference to studies by Cheng and Stough [44], the average probability elasticity was calculated to assess the magnitude of estimated coefficients. Here, the elasticity of industry-specific agglomeration of CODI (*Specialized_CN*), 0.233, means that a 10% increase of previous CODI stock of a specific industry in a state leads to a 2.33% increase in the probability that CODI of the same industry will choose that particular state on average. As Table 4 indicates, the elasticity of *Specialized_CN* (0.233) is larger than *Diversified_CN* (0.085), suggesting that regarding the influence of previous CODI stock, although both industry-specific and industry-diverse agglomeration of Chinese investors play a positive role in the location choice of CODI, the industry-specific agglomeration of CODI is more instructive. Therefore, H2 is verified. Furthermore, it is noteworthy that the elasticity of *Local specialized agglomeration* (0.340) is larger than *Specialized_CN* (0.233), suggesting that the attractive effect of the specialized agglomeration of local firms surpasses the industry-specific agglomeration of CODI of the same industry. This finding is in line with the study by Lee et al. [45], who found that when deciding on the focal state in the United States, Korean multinational enterprises are more sensitive to the agglomeration of local firms than to the agglomeration of Korean firms, suggesting that the impact of factor endowments exceeds that of nationality. This may also imply that in a specific industry, CODI is more sensitive to the knowledge spillovers resulting from the local firms of the United States rather than from the CODI stock in the same state, especially in the context of Chinese investors following learning-based knowledge-seeking strategies [3, 46].

Regarding the results of the control variables, Overseas Chinese do influence the location choice of CODI, which corresponds to past studies [8, 38]. The number of patents of the states also has positive effects on the location decision, proving that Chinese investors are seeking strategic resources. Higher taxes also lead to lower CODI, to some extent implying that the United States market is an important factor that affects the location choice of CODI. However, natural resources have a significantly negative impact on attracting CODI, possibly because the expense of resource-seeking CODI is too high in the United States.

Furthermore, as Table 5 showed, four patterns of agglomeration economies have varied influences on the location choice of CODI in the context of different entry modes and ownerships. Firstly, as shown in Model 5, the elasticity of *Local specialized agglomeration* (0.409) in the context of acquisition investment is larger than that of greenfield investment. One plausible reason is that Chinese investors can quickly gain strategic resources through acquisition [31], resulting in more focus on the specific industry knowledge from the local firms. This inference is also evidenced by Chinese transnational acquirers focusing more on the greater elasticity of the number of patents of the states and significantly lower R&D expense. In addition, in contrast to greenfield investment, the location choice of acquisition is not sensitive to the industry-diverse agglomeration of CODI. Moreover, the growth rate of the GDP of the states has a significantly positive impact on attracting CODI, revealing Chinese transnational acquirers’ attention to the market [47].

**Table 5:** Conditional logit estimation for CODI in the United States according to entry modes and ownerships.

	Model 5				Model 6			
	Greenfield		Acquisition		Private firms		SOE	
Variable	Coefficient	Elasticity	Coefficient	Elasticity	Coefficient	Elasticity	Coefficient	Elasticity
*Local specialized agglomeration*	0.284*** (0.061)	0.278	0.417*** (0.064)	0.409	0.276*** (0.052)	0.271	0.414*** (0.086)	0.406
*Local diversified agglomeration*	0.016 (0.050)	0.016	0.003 (0.062)	0.003	-0.021 (0.044)	-0.021	0.105 (0.077)	0.103
*Specialized_CN*	0.259*** (0.035)	0.254	0.201*** (0.038)	0.197	0.212*** (0.029)	0.208	0.371*** (0.060)	0.364
*Diversified_CN*	0.121*** (0.036)	0.119	0.042 (0.042)	-0.041	0.091*** (0.031)	0.089	0.025 (0.059)	0.025
*Overseas Chinese*	0.445*** (0.089)	0.436	0.450*** (0.121)	0.441	0.411*** (0.853)	0.403	0.563*** (0.148)	0.552
*Patents*	0.439*** (0.078)	0.430	0.575*** (0.100)	0.564	0.557*** (0.072)	0.546	0.341*** (0.120)	0.334
*R&D expense*	-0.009 (0.052)	-0.009	-0.133** (0.059)	-0.130	-0.049 (0.046)	-0.048	-0.079 (0.084)	-0.077
*Labour quality*	-0.043 (0.033)	-0.042	-0.002 (0.035)	-0.002	-0.038 (0.030)	-0.037	-0.008 (0.039)	-0.008
*Growth_GDP*	-0.005 (0.027)	-0.005	0.101*** (0.032)	0.099	0.034 (0.026)	0.033	0.057* (0.031)	0.056
*Tax*	-1.611*** (0.375)	-1.579	-0.360 (0.431)	-0.353	-1.095*** (0.328)	-1.074	-1.597*** (0.495)	-1.566
*Income*	0.763 (1.068)	0.748	-0.525 (1.337)	-0.515	-0.216 (1.018)	-0.212	1.928 (1.322)	1.890
*Resource*	-6.169*** (2.368)	-6.048	-6.029** (2.546)	-5.911	-9.469*** (1.859)	-9.283	0.971 (2.870)	0.952
Number of observations	3811		3216		5386		1641	
Pseudo R^2^	0.403		0.497		0.444		0.468	
Log pseudolikelihood	-682.635		-484.991		-897.474		-261.850	

Notes: In the practical process of using Stata, we use "robust" command to account for potential model misspecification. Robust Standard errors in parentheses.

*** p<0.01,

** p<0.05,

* p<0.1.

With respect to the choice difference of the ownerships, as Model 6 shows, compared to the private CODI, the location choice of SOEs’ offshoring subsidiaries is more sensitive to the United States firms and industry-specific agglomeration of CODI than that of private investment, and the industry-diverse agglomeration of CODI has no significant impact on the location choice of SOEs’ offshoring subsidiaries. The rationale behind this finding may be that the strategic need of SOEs to acquire unique resources and capabilities [48] leads to the focus on the specific industry agglomeration. In addition, private investors tend to invest more in the states with more patents than state-owned investors, while being reluctant to locate in the states with abundant natural resources.

## 5. Conclusion and discussion

Based on the data of the US-China Investment Project, we use a conditional model to investigate the influence of different patterns of agglomeration economies on the location choice of CODI in the United States in the period 2000–2016. Our study confirms that agglomeration economies do influence the location decision of CODI. The specialized agglomeration of local firms exerts more influence on the location choice of CODI than industry-specific agglomeration of CODI in the same industry. Industry-diverse agglomeration of CODI plays a weak role, while diversified agglomeration of local firms has no effect. Moreover, the heterogeneity of firms in terms of the entry mode and ownership also impacts the influence of agglomeration economies on the location decision of CODI. The location choice of acquisition is more sensitive to the specialized agglomeration of local firms than that of greenfield investment, while the influence of the industry-diverse agglomeration of CODI has no effect on the location choice of acquisition. The location choice of SOEs’ offshoring subsidiaries is more sensitive to specialized agglomeration of local firms and industry-specific agglomeration of CODI than that of private firms, and the influence of the industry-diverse agglomeration of CODI has no effect on the location choice of SOEs’ offshoring subsidiaries.

Under the Belt and Road Initiative, more Chinese firms are going abroad to participate in economic globalization. In this case, this paper can contribute impulses for CODI in three ways. Firstly, it expounds which types of agglomeration play a role in the location selection of CODI and shows the difference in the influence of the agglomeration with respect to the entry mode and ownership, which not only responds to the call by Head et al. [20] to account for different patterns of agglomeration in the location decision, but also provides reference for CODI to make location decisions from the perspective of industrial agglomeration. Secondly, this paper focuses on the location choice of CODI in different regions within a country and provides empirical evidence for the research on the location choice of CODI at a more detailed level. Thirdly, this paper reveals the current location choices of CODI in the United States and provides reference for subsequent CODI there. In addition, this study may have the following policy implications: firstly, the government needs to consider the differences of the invested regions’ factor endowments when formulating the incentive policies for investing abroad in order to better integrate global resources; secondly, the government should formulate more specific investment policies based on the ownership of Chinese multinational enterprises in order to encourage them to actively participate in foreign direct investment.

This study confirms that the influence of different patterns of agglomeration economies on the location choice of CODI in the United States varies, however it does not address an important question, namely the sources of agglomeration economies affecting the location choice of CODI. The heterogeneity of industry of the firms also impacts the agglomeration economies in their location decision, an issue that has also not been solved in this paper. Further research is recommended to investigate these questions in order to better understand the location choice of CODI. Moreover, due to different investment behaviours of CODI in developed and developing countries, more research must also be conducted to determine whether the findings of this paper can be applied to other countries.
